# Generalized Myoclonus in an Elderly Patient on Risperidone: A Rare but Important Adverse Effect

**DOI:** 10.1155/crps/7576076

**Published:** 2025-07-18

**Authors:** Özlem Totuk, Özge Gönül Öner

**Affiliations:** ^1^Neurology, Sancaktepe Sehit Prof. Dr. Ilhan Varank Research and Training Hospital, Istanbul, Türkiye; ^2^Neurology, Göztepe Prof. Dr. Süleyman Yalçın City Hospital, Istanbul, Türkiye

**Keywords:** adverse effect, drug-induced myoclonus, risperidone, serotonin syndrome, sertraline

## Abstract

**Objective:** Drug-induced myoclonus is a rare adverse effect associated with several drug classes, including antipsychotics. This case report describes a patient who developed generalized myoclonic jerks while on long-term risperidone therapy, in the context of concomitant sertraline use, without fulfilling clinical criteria for serotonin syndrome.

**Case Presentation:** We present a 73-year-old male patient with a history of cerebrovascular disease and behavioral symptoms treated with sertraline and risperidone. The patient developed sudden, generalized myoclonic movements. Neurological examination and imaging studies excluded acute structural pathology and metabolic or infectious causes. Serotonin syndrome was ruled out based on clinical findings. The myoclonus resolved rapidly after discontinuation of risperidone, without any specific symptomatic treatment, suggesting a potential drug-related mechanism.

**Conclusion:** This case highlights the importance of recognizing antipsychotic-related myoclonus as a potentially reversible condition. Clinicians should consider drug-induced myoclonus in the differential diagnosis and pay special attention to the risks posed by polypharmacy involving psychotropic agents.

**Summary**

• Drug-induced myoclonus is a rare but treatable condition.
• Risperidone may cause generalized myoclonus independent of serotonin syndrome.
• Discontinuation of the drug resulted in full recovery.
• Clinicians should consider medication side effects in new-onset movement disorders.

## 1. Introduction

Myoclonus is characterized by involuntary, brief, and sudden movements resulting from muscle contractions or, less commonly, inhibitions of muscle activity, which manifest as negative myoclonus [1]. The etiology of myoclonus can be divided into four main categories: physiological, essential, epileptic, and secondary (symptomatic) causes [2]. Symptomatic myoclonus accounts for 72% of all myoclonus cases, including drug-induced myoclonus and toxic syndromes [3]. Drug-induced myoclonus, a rare condition, falls within the symptomatic category [4].

Risperidone is an atypical antipsychotic with potent 5HT2 and D2 receptor antagonism, primarily used in the treatment of schizophrenia. While myoclonus triggered by atypical antipsychotics is rare, risperidone-induced myoclonus is even rarer, with only a single case report available describing risperidone-induced myoclonus independent of serotonin syndrome [5, 6].

## 2. Case Presentation

A 73-year-old man was admitted to the emergency department with complaints of sudden-onset, involuntary jerking movements affecting the whole body and extremities, which had started the previous day (Supporting Information Video 1). The distinction between epileptic and nonepileptic movements was made based on simple observation. His medical history included hypertension and cerebrovascular disease, without any history of epilepsy. He had been receiving sertraline 100 mg/day and risperidone 1 mg/day for approximately 4 years, which were initiated by the psychiatry department for behavioral disturbances, although no formal psychiatric diagnosis was available in the medical records. He was also taking acetylsalicylic acid 100 mg/day for cerebrovascular prophylaxis.

On examination, he had frequent, generalized, irregular, and brief jerking motions. Cognitive assessment revealed an Addenbrooke's Cognitive Examination-Revised III score of 33/100, indicating significant impairment in memory, attention, fluency, language, and visuospatial abilities [7]. There was no history suggestive of overdose, and drug adherence was confirmed through caregiver reporting.

His neurological examination was otherwise normal, apart from gait instability related to the involuntary movements. Body temperature remained stable (36.2°C), and there was no evidence of autonomic dysfunction or altered mental status. Electroencephalography (EEG) was normal despite clinical myoclonus. Brain magnetic resonance imaging (MRI) showed age-related atrophy and white matter ischemic changes, without acute pathology.

Laboratory investigations revealed mildly elevated white blood cell count (11.8 × 10^9^/L) and stable renal function (blood urea nitrogen 24 mg/dL and creatinine 1.62 mg/dL). Liver and thyroid function tests, creatine kinase, blood and urine cultures, and thoracic computed tomography were unremarkable.

In the absence of infectious, metabolic, or structural abnormalities, drug-induced secondary myoclonus was considered. Serotonin syndrome was ruled out based on the lack of mental status changes and autonomic symptoms. Following psychiatric consultation, risperidone was discontinued, leading to complete resolution of the involuntary movements within 24 h. The patient was discharged without recurrence 4 days later, confirming a diagnosis of risperidone-induced myoclonus.

## 3. Discussion

The exact mechanism of drug-induced myoclonus is not fully understood, but it generally resolves upon discontinuation of the causative agent [8]. Myoclonus has been associated with various drug classes, including antiparkinsonian agents (e.g., levodopa), psychiatric medications (e.g., selective serotonin reuptake inhibitors, monoamine oxidase inhibitors, and lithium), antibiotics (e.g., penicillins, cephalosporins, and quinolones), narcotics, anticonvulsants, anesthetics, and cardiac drugs, such as calcium channel blockers and antiarrhythmics [9]. Withdrawal from sedative agents is also a known cause.

In this case, both risperidone and sertraline were considered potential contributors, as they are independently capable of inducing myoclonus and may act on overlapping serotonergic and dopaminergic pathways. Due to the patient's behavioral symptoms, both drugs could not be discontinued simultaneously. Following the withdrawal of risperidone alone, the myoclonic jerks resolved completely within 24 h, strongly suggesting risperidone as the primary agent.

Although serum levels of risperidone and sertraline were not measured, their absence does not preclude clinical inference. A synergistic interaction between the two drugs may have contributed to symptom development; however, complete resolution with risperidone withdrawal supports its predominant role. Furthermore, serotonergic, dopaminergic, and GABAergic mechanisms are believed to underlie antipsychotic-induced myoclonus [10]. It is also notable that approximately 25% of patients using antipsychotics for extended periods may develop tardive myoclonus, a delayed-onset, postural form of the disorder [11].

## 4. Conclusion

This case highlights that, although rare, atypical antipsychotics, such as risperidone, can lead to serious adverse effects, including myoclonus. Clinicians should consider antipsychotic-induced myoclonus in the differential diagnosis of new-onset movement disorders and perform a thorough review of current pharmacotherapy. In most instances, discontinuation of the offending agent leads to complete symptom resolution.

Particular caution is advised when multiple psychotropic agents are prescribed concurrently, as potential synergistic interactions may elevate the risk of significant neurological complications.

## Data Availability

No datasets were generated or analyzed during the current study.

## Supporting Information

Supporting Information A Supporting Information video file has been submitted, which shows the generalized myoclonic movements observed in the patient during clinical evaluation prior to risperidone withdrawal. The video supports the diagnostic impression and has been anonymized to protect patient identity.
